# A Case of Euthyroid Graves' Ophthalmopathy in a Patient Sero-Negative for TSH Receptor Autoantibody

**DOI:** 10.1155/2018/1707959

**Published:** 2018-06-13

**Authors:** Asami Hotta, Tomohiro Tanaka, Haruka Kato, Shota Kakoi, Yuki Shimizu, Chie Hasegawa, Akiko Hayakawa, Satoshi Yasuda, Kento Ogawa, Shunsuke Ito, Hideomi Ohguchi, Takashi Yagi, Hiroyuki Koyama, Mihoko Kawamura, Kazuhiko Sugitani, Yuichiro Ogura, Takashi Joh, Kenro Imaeda

**Affiliations:** ^1^Department of Gastroenterology and Metabolism, Nagoya City University Graduate School of Medical Sciences, 1 Kawasumi, Mizuho-cho, Mizuho-ku, Nagoya 467-8601, Japan; ^2^Department of Ophthalmology, Inazawa Kosei Hospital Aichi Prefectural Welfare Federation of Agricultural Cooperatives, 7 Jicchouno-Honkou, Sobue-cho, Inazawa, Japan; ^3^Department of Ophthalmology, Nagoya City University Graduate School of Medical Sciences, 1 Kawasumi, Mizuho-cho, Mizuho-ku, Nagoya 467-8601, Japan; ^4^Department of Endocrinology and Diabetes, Nagoya City West Medical Center, 1-1-1, Hirate-cho, Kita-ku, Nagoya 462-8508, Japan

## Abstract

We report of a case of Graves' ophthalmopathy presented solely with symptoms of the eyes with normal thyroid function tests and negative immunoreactive TSH receptor autoantibody. 40-year-old male was referred to our hospital due to 2-month history of ocular focusing deficit without any signs or symptoms of hyper- or hypothyroidism. Serum thyroid function tests and ^99m^Tc uptake were both within the normal range. Anti-thyroid autoantibodies were all negative except for the cell-based assay for serum TSH receptor stimulating activity. Since orbital CT scan and MRI gave typical results compatible with Graves' ophthalmopathy, we treated the patients with corticosteroid pulse therapy and orbital radiation therapy, leading to a partial improvement of the symptoms. This case gives insights into the potential pathophysiologic mechanism underlying Graves' ophthalmopathy and casts light upon the difficulties of establishing the diagnosis in a euthyroid case with minimal positive results for anti-thyroid autoantibodies.

## 1. Introduction

Graves' ophthalmopathy is a potentially sight-threatening disease of the orbital cavity, generally accompanied by hyperthyroidism associated with Graves' disease [1]. However, the diagnosis of Graves' ophthalmopathy has always posed challenges for endocrinologists, because, in some cases, results of the thyroid function test remain normal or even lower than the reference levels [1].

We have experienced a case of euthyroid Graves' ophthalmopathy presenting with negative thyroid autoantibodies except TSH stimulating antibodies (TSAb). Since the clinical establishment of the diagnosis of Graves' ophthalmopathy in this case was arduous, we here present the case with a comprehensive review of the literature of TRAb-negative, euthyroid Graves' ophthalmopathy.

## 2. Case Presentation

40-year-old male patient was referred to our department with two-month history of ocular focusing deficit without any signs or symptoms suggestive of thyroid dysfunction. Past illness or family history did not reveal any presence of thyroid-related diseases. He has not been taking any medication but has consumed 20 cigarettes a day for 20 years.

Ophthalmological examination has revealed double vision on upward gaze with disturbance in upward movement of the left eye, eyelid retraction, and exophthalmos of the left eye. Intraocular pressure and visual acuity were normal. The exophthalmoses on the right and left sides were 15mm and 19mm by Hertel exophthalmometer (normal range: 10~15mm with laterality of less than 3mm for the Japanese). Clinical activity score (CAS) of the ophthalmopathy was 2 with redness and swelling of the eyelid.

Although no physical sign of thyroid dysfunction was observed, thyroid function tests were performed since Graves' ophthalmopathy was suspected. Plasma FT3, FT4, and TSH levels were as 2.75pg/mL, 1.38ng/dL, and 0.934*μ*IU/mL, respectively, and were within the normal range (Table 1). Thyroid peroxidase antibodies (TPOAb), thyroglobulin antibodies (TgAb), and TSH receptor autoantibodies (TRAb) were all negative. Only TSAb was slightly positive: 146% (normal range ≦120%) (Table 1). Rheumatoid factor was negative and Immunoglobulin (Ig) G, IgA, and IgM levels were all within normal range. Although C3 or C4 levels were also within normal range, CH50 was slightly higher (59.4 U/mL) than the normal range (32-48 U/mL). Kidney and liver functions were within normal limit. HBV, HCV, HTLV-1, and HIV were negative.

**Figure 1:**
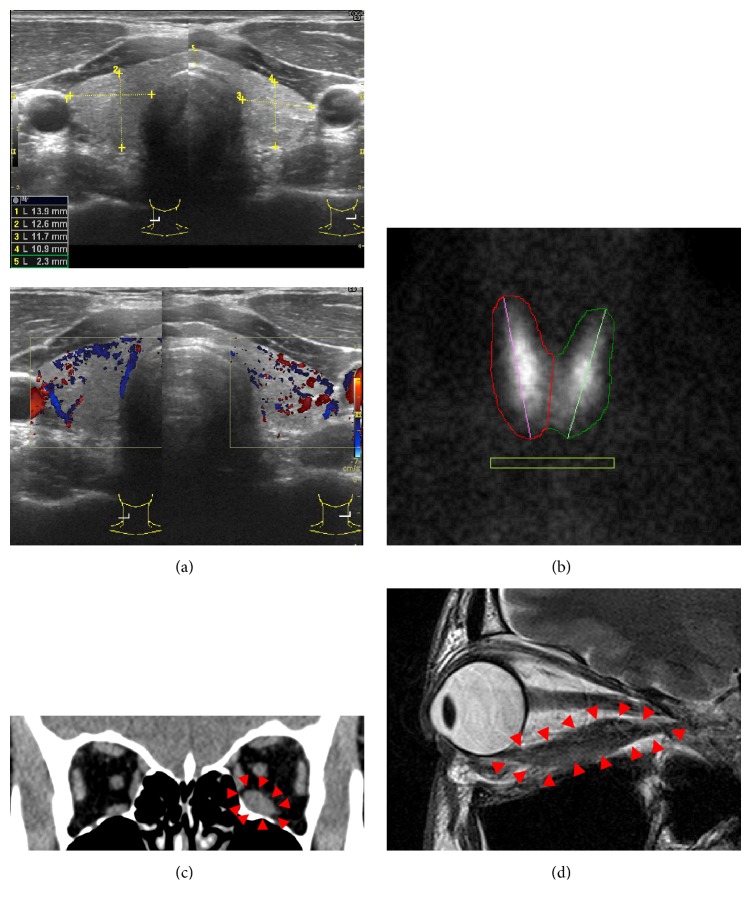
Imaging studies. (a) Ultrasonographic images of the thyroid showing normal-sized gland with slightly enhanced blood flow. (b) ^99m^Tc scintigraphy exhibiting an uptake within the normal range. (c) CT scan image showing enlarged inferior rectus muscle of the left eye. (d) Fat-suppression T2-weighted MRI image of the left eye showing inferior rectus muscle swelling with slightly high intensity signal of the muscle body.

Ultrasonography of the thyroid gland was performed. It showed normal-sized gland with slightly enhanced blood flow (Figure 1(a)). To directly measure thyroid activity* in vivo*, ^99m^Tc scintigraphy was then performed. The result was again negative with ^99m^Tc uptake of 0.50% for the right gland, 0.39% for the left gland, and total 0.89% (normal <5% in total) (Figure 1(b)). These serological and imaging studies of the thyroid has scarcely given positive evidence for the diagnosis of Graves' disease in this patient.

We next performed imaging analysis of the orbital cavity. CT scan showed obvious enlargement of the inferior rectus muscle of the left eye (Figure 1(c)). MRI images of the orbit again showed inferior rectus muscle swelling of the left eye (Figure 1(d)). Fat-suppression T2-weighted images showed a slight increase in the MR intensity of the muscle involved (Figure 1(d)), suggesting active inflammation of the extraocular muscle. No tumorous lesion was detected within the orbital cavity (Figures 1(c) and 1(d)).

With these findings, the differential diagnosis of this case were (1) euthyroid Graves' ophthalmopathy with a slight increase in TSAb alone or (2) idiopathic inflammation of the extraocular muscle [2]. Based on the insidious onset, absence of ocular pain, predominance of inferior rectus muscle swelling, and apparent muscular swelling with minimally swollen tendons [1–3], we diagnosed this case as TRAb-negative euthyroid Graves' ophthalmopathy.

**Figure 2:**
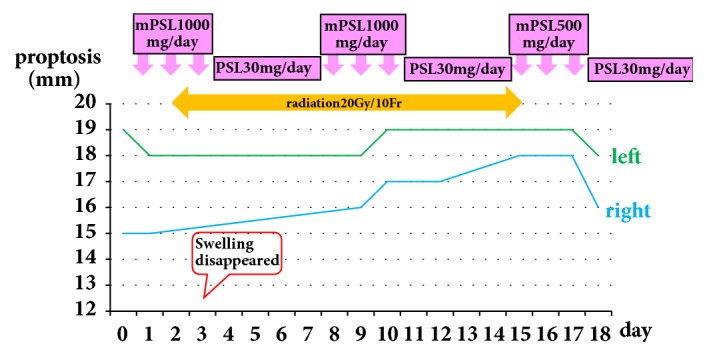
Clinical course of the patient. The patient was hospitalized and received intravenous and oral corticosteroid therapy in conjunction with orbital radiotherapy. Intravenous methylprednisolone 1000mg/day for 3 days followed by oral prednisolone 30mg/day for 4 days were defined as one cycle, and he received 3 cycles consecutively with a slightly decreased methylprednisolone i.v. in the third cycle. Orbital radiation therapy was commenced on day 2 and 2Gy/day radiation was continued for 10 days (20Gy total). The patient was discharged on oral corticosteroid therapy with decreasing doses.

Under the diagnosis, he was hospitalized and treated with the combination of corticosteroid pulse therapy and radiation therapy of the orbit (Figure 2). Intravenous administration of methylprednisolone 1000mg/day for 3 days followed by oral prescription of prednisolone 30mg/day for 4 days were undertaken as the first cycle of the medical therapy. He underwent this medical therapy for consecutive three cycles with a slight modification of decreased methylprednisolone dose (500mg/day) in the third cycle due to a slight elevation of the liver function tests. After the third cycle of the therapy, he was given oral prednisolone as an out-clinic patient. The corticosteroid doses were decreased by 5mg every two weeks until cessation. On the second day of the first cycle of the corticosteroid pulse therapy, daily orbital radiation therapy of 2Gy/day was initiated and performed for ten days (20Gy total). Although the swelling of the left eyelid disappeared on day 3 (Figure 2), proptosis, abnormality in ocular movement, and diplopia were all unchanged until discharge. Examination by the exophthalmometer revealed the remaining of the exophthalmoses of 16mm on the right and 18mm on the left when he left hospital on day 18 (Figure 2). Thyroid function remained within the normal range throughout the clinical course and TSAb turned negative (113%) four months after the initiation of corticosteroid therapy.

## 3. Discussion

Here we report of a euthyroid case presented with symptoms, signs, and imaging results typical of Graves' ophthalmopathy but with negative TRAb. Although TSAb in this case was slightly positive, suggesting the involvement of autoimmune etiology, establishing the definitive diagnosis of Graves' ophthalmopathy was difficult. In this report we summarize our current case and review reported euthyroid cases of Graves's ophthalmopathy presenting with negative autoantibody.

One of the postulated hypotheses is that, in Graves' ophthalmopathy, proteins derived from orbital fibroblasts serve as autoantigens and that autoimmune responses against these autoantigens play a pivotal role in the pathogenesis of the disease [1]. It has been suggested that some subpopulations of these orbital fibroblasts or its offspring orbital adipocytes also take part in the inflammatory process by expressing “ectopic” thyrotropin receptor [4]. Another mechanism suggested in Graves' ophthalmopathy is that the autoantigen called calsequestrin derived from ocular muscles serves as a target of autoimmunity [5]. Although its pathogenic role remains under debate, calsequestrin has emerged as a new sensitive and specific biomarker of ophthalmopathy in patients with Graves' disease [6, 7].

The triggering event of autoimmune reaction in Graves' ophthalmopathy, however, has not been fully elucidated [1]. Furthermore, the reason why thyroid gland, orbital tissue including fibroblasts, and ocular muscle cells and sometimes skin are the selected targets of autoimmunity in Graves' ophthalmopathy or dermopathy has not been addressed [1]. Potential similarities among the antigenic properties of these three tissues, such as the shared expression of thyrotropin receptor, have been suggested [8]. Since reactive lymphocytes involved in autoimmunity should be polyclonal by nature, it is tempting to speculate that the spectra of autoantigens recognized by the autoantibodies present in Graves' disease may differ from case to case. Differences in antigen repertoires among patients with Graves' disease may partly explain variations in clinical severity of the orbital disease.

In our case, TSAb was positive. Our assay is based on cAMP production by filtered porcine primary thyroid cell clusters in response to stimulation by TSH-deprived human sera. When 120% is set as the cut-off value, sensitivity and specificity of the test are 99.1% and 100.0%, respectively [9]. However, TRAb was negative and thyroid function was normal. Considering the possible antigenic variations, autoantibodies in this patient may be targeted more specifically to orbital autoantigens and not to follicular cells of the thyroid. Or otherwise, TSAb produced in this patient may have acted on the thyroid cells and upregulated the expression or exposure of antigens, which is only barely found in normal thyroid tissues, but abundantly expressed in orbital tissues [10]. However, further immunological analyses using patient sera are warranted to elucidate potential mechanisms linking orbital disease and thyroid pathology in this patient. Graves' ophthalmopathy is common in Europeans [11]. Some potential polymorphisms in the genes essential for the autoimmune response, either on the antigen side or on the immune cell side, may account for the apparent higher incidence of this disease in the European population.

In our case, the differential diagnosis should be as follows: (1) intraorbital malignancies, (2) granulomatous diseases such as Wegener's granulomatosis or sarcoidosis, and (3) idiopathic orbital inflammatory disease [12]. No involvement of the other organs or tissues, e.g., uveitis, hilar lymph node swelling, heart, lung, kidney, or skin lesions in this case, argues against the diagnosis of Wegener's granulomatosis or sarcoidosis. Malignant tumor has not been suggested because the imaging studies showed only the limited swelling of the inferior rectus muscle without any intraorbital space occupying lesion.

The clinical features of the idiopathic inflammation of the extraocular muscle also known as idiopathic orbital myositis are acute and severe pain of the eyes, proptosis, eyelid swelling, and erythema, and conjunctival redness and chemosis [2, 13]. MRI images of the disease reportedly show unilateral enlargement of the extraocular muscle (especially superior rectus or lateral rectus muscles) that involves the tendons with vague contour of the muscle [3, 13]. In the current case, the absence of ocular pain, conjunctival redness, or chemosis, the predominance of inferior rectus muscle swelling with minimal involvement of the tendons, made us negate the possibility of the idiopathic inflammation of the extraocular muscle. As such, although TRAb was negative and TSAb only slightly positive, we diagnosed this case as euthyroid Graves' ophthalmopathy. However, since slightly high CH50 level was the only hint of inflammatory reaction in this patient and no autoantibody tested was strongly positive, there remains a possibility that the pathology in this patient is not autoimmunity in nature. If we could test the potential recognition of orbital muscle cells or connective tissue by this patient's serum, we might be more convinced of the involvement of autoimmune mechanisms in the pathogenesis of ophthalmopathy.

In a report, the thyroid function at manifestation was either euthyroid or hypothyroid in 4.2 percent of all the patients with Graves' ophthalmopathy [14]. In general, about 10% of the patients with Graves' ophthalmopathy remain euthyroid or develop hypothyroidism during the clinical course [15]. Some cases even sero-negative for TRAb assay have been reported [16–20]. In fact, up to 5% of euthyroid or hypothyroid patients with Graves' ophthalmopathy reportedly exhibit low titers for anti-thyroid autoantibodies [1]. In these cases, efforts have been made to obtain at least one or more positive results in autoantibody screening. Among these, some cases were positive for TSAb and some for thyroid-stimulating immunoglobulins (TSI). TSAb bioassay was performed and was positive in two Japanese patients with Graves' ophthalmopathy who were TRAb-negative and euthyroid [16]. In one of the two cases, lateral and inferior rectus muscles of the right eye were swollen with pain, lid retraction, proptosis, and diplopia [16]. In the second case, medial rectus muscle swelling, lid retraction, and proptosis of the right eye and diplopia were observed [16]. TSAb in these two cases were 508% and 240%, respectively [16]. In the paper, the authors discuss that it is an advantage of TSAb over TRAb for the more accurate diagnosis of Graves' ophthalmopathy [16]. Similarly, weakly positive result for TSAb in our case enabled us to be more confident in our diagnosis of Graves' ophthalmopathy. Without this positive TSAb result, the diagnosis would have been more difficult.

Treatment of choice for moderately to severely active Graves' ophthalmopathy is intravenous corticosteroid therapy and orbital radiation therapy [15]. Some reports have suggested the effectiveness of intravenous corticosteroid therapy also for euthyroid and TRAb-negative Graves' ophthalmopathy [16]. In the present case, however, intravenous corticosteroid therapy and adjunct orbital radiation therapy led to an improvement of the eyelid swelling alone and was not effective for proptosis, leaving double vision still symptomatic. Ophthalmologist later proposed him a surgical treatment option, which the patient declined. Since reports have suggested a future possibility of the development of hyperthyroidism and/or positive TRAb test [15], the case has been regularly followed until present without any worsening.

## 4. Conclusion

We report a case of euthyroid Graves' ophthalmopathy with negative TRAb and slightly positive TSAb autoantibody. The diagnosis was made by excluding other causes of extraocular orbital muscle swelling such as granulomatous diseases, malignant tumors and idiopathic myositis. In such a case with minimal positive results for anti-thyroid autoantibodies, the diagnosis of Graves' ophthalmopathy is an arduous process requiring careful review of the medical records and imaging results.

## Figures and Tables

**Table 1:** Laboratory data at presentation.

test	result	normal range
WBC	9.8	3.6~9.6 × 10^∧^3/*μ*L
Hb	16.1	13.2~17.2 g/dL
PLT	271	148~339 × 10^∧^3/*μ*L
CK	120	62~287 U/L
AST	17	13~33 U/L
ALT	22	6~30 U/L
ALP	207	115~359 U/L
Glucose	105	70~109 mg/dL
T-chol	190	128~219 mg/dL
LDL	122	<120 mg/dL
ACE	10.1	8.3~21.4 U/L
TSH	0.934	0.340~4.220 *μ*IU/mL
FT4	1.38	0.77~1.59 ng/dL
FT3	2.75	2.24~3.94 pg/mL
thyroglobulin	6.57	≦33.7 ng/mL
TRAb (CREIA)	1.47	<2.0 IU/L
TPOAb (CREIA)	2.2	<9.4 IU/mL
TgAb (CREIA)	10.0	≦54.6 IU/mL
TSAb	146	≦120%
